# Cumulative lifetime stressor exposure and health in elite athletes: the moderating role of perfectionism

**DOI:** 10.1080/1612197x.2022.2153203

**Published:** 2022-12-28

**Authors:** Ella McLoughlin, David Fletcher, Hannah L. Graham, Rachel Arnold, Daniel J. Madigan, George M. Slavich, Lee J. Moore

**Affiliations:** a Department for Health, University of Bath, Bath, UK;; b School of Sport, Exercise and Health Sciences, Loughborough University, Loughborough, UK;; c School of Science and Technology, Nottingham Trent University, Nottingham, UK;; d School of Science, Technology, and Health, York St John University, York, UK;; e Department of Psychiatry and Biobehavioral Sciences, University of California, Los Angeles, CA, USA

**Keywords:** Life stress, adversity, mental health, physical health, sport

## Abstract

Although greater lifetime stressor exposure has been associated with physical and mental health issues in the general population, relatively little is known about how lifetime stressors impact the physical and mental health of elite athletes or the factors moderating this association. Given that many elite athletes show signs of perfectionism, and that this trait has been linked with ill-health, it is possible that perfectionism may moderate the lifetime stressor-health relationship. To test this possibility, we examined how cumulative lifetime stressor exposure was associated with general mental and physical health complaints in elite athletes, and the extent to which these associations were moderated by perfectionism. Participants were 110 elite athletes (64 female; *M*_*age*_= 29.98 years, *SD* = 10.54) who completed assessments of lifetime stressor exposure, physical health, psychological distress, and perfectionism. As hypothesised, hierarchical regression analyses revealed that experiencing more severe lifetime stressors was related to poorer physical and mental health. Furthermore, self-oriented perfectionism moderated the association between lifetime stressor count and severity and physical health, but not mental health. Overall, these data demonstrate stressor-specific effects among elite athletes and highlight the potential importance of assessing lifetime stressor exposure and perfectionistic tendencies in order to improve athlete health and well-being.

Research suggests that lifetime stressor exposure is an important predictor of a variety of health-related outcomes (McLoughlin et al., 2022; Slavich & Shields, 2018). Recent work on this topic has used the Stress and Adversity Inventory for Adults (STRAIN) to assess exposure to major stressors occurring over the entire life course. The STRAIN systematically enquires about a stressor’s type (e.g., acute life events vs. chronic difficulties), exposure timing (e.g., early life vs. adulthood), primary life domain (e.g., education, work), and social-psychological characteristic (e.g., interpersonal loss, physical danger), and thus provides a comprehensive picture of an individuals’ stressor exposure. Since its initial development, the STRAIN has been used to examine how lifetime stressor exposure predicts numerous biological, behavioural, and clinical outcomes (e.g., Alvarez et al., 2019; Pegg et al., 2019). Broadly speaking, this research demonstrates that as the frequency and severity of lifetime stressors increases, so too does the vulnerability to future psychological problems (e.g., depression and anxiety; Pegg et al., 2019; Slavich et al., 2019), as well as physical health complaints (e.g., colds, coughs; Cazassa et al., 2020). As a result, it is relatively well established that lifetime stressor exposure contributes to various health problems in the general population (Slavich, 2016). However, research on this topic is limited among sport performers, particularly, elite athletes who experience high levels of stress-related burden (c.f. McLoughlin et al., 2021).

Initial research within a sporting context has suggested that elite athletes who have experienced greater lifetime (non-sport) stressors report greater symptoms of depression and anxiety, as well as lower levels of psychological well-being (McLoughlin et al., 2021). Moreover, this research has revealed that chronic difficulties (vs. acute life events) and adulthood (vs. early life) stressors were particularly detrimental to elite athletes’ health and well-being (McLoughlin et al., 2021). Additionally, the qualitative findings reported by McLoughlin et al. (2021) suggested that relatively high lifetime stressor exposure fostered poorer health and well-being by promoting greater use of maladaptive long-term coping strategies (e.g., denial, avoidance coping), increased susceptibility to future stress, and limiting interpersonal relationships (i.e., social support; McLoughlin et al., 2021). More recently, these results were extended by McLoughlin et al. (2022), who found that greater lifetime (non-sport and sport-specific) stressor exposure was associated with more mental and physical health problems (e.g., depression, respiratory infections). The results revealed that sport performers who experienced more severe lifetime (non-sport and sport-specific) stressors were more likely to appraise potentially stressful situations as a threat (i.e., situational demands exceed coping resources) than a challenge (McLoughlin et al., 2022). Despite improving our understanding of stress-health associations and possible mediators (e.g., appraisals) among athletes, this research did not explore potential moderating factors, such as personality. This is surprising given the prominent role that personality is proposed to play in stress-health theories, such as the integrative model of stress and health (Epel et al., 2018).

Given the detrimental consequences that greater lifetime stressor exposure can have on health-related outcomes, it is essential to identify factors that may increase one’s vulnerability or resiliency to stress-related diseases (Epel et al., 2018). Although there is an abundance of moderators that could be examined, one characteristic that has received growing attention and appears to be increasing in prevalence is perfectionism (Curran & Hill, 2019; Hill et al., 2018). At its broadest, perfectionism is a multidimensional personality trait that is characterised by a combination of striving for flawlessness and overly critical evaluations of behaviour (Frost et al., 1990). One of the most utilised models of perfectionism was proposed by Hewitt and Flett (1991a), which differentiates between three dimensions of perfectionism based on the direction of perfectionistic thoughts, feelings, and behaviours. The first dimension, self-oriented perfectionism, is the demand of perfection from the self. The second dimension, socially prescribed perfectionism, is the belief that others expect one to be perfect. And the final dimension, other-oriented perfectionism, is the demand of perfection from others (Hewitt & Flett, 1991a). Importantly, these different dimensions are thought to have varying effects on outcomes such as performance and health (Molnar et al., 2012).

Some investigators have suggested that perfectionism is essential to obtain success in sport (e.g., Hardy et al., 2017). However, research has also examined perfectionism in relation to physical and mental health in both general and clinical populations (Sirois & Molnar, 2016). In this regard, there is evidence that the direction, size, and strength of associations differs based on which dimension is examined. Generally, socially prescribed perfectionism has shown the strongest positive associations with mental health outcomes such as depression and disordered eating (e.g., Limburg et al., 2016), as well as aspects of physical health (e.g., migraines, gastrointestinal illnesses, and hypertension; Flett et al., 2016). Similarly, other-oriented perfectionism is positively related to health outcomes, but the effects are typically smaller in size (e.g., Saboonchi & Lundh, 2003). Finally, the association between self-oriented perfectionism and health is more ambiguous, with some studies showing positive associations and others revealing negative associations (e.g., Molnar et al., 2012). These mixed findings reflect self-oriented perfectionism’s potential to energise behaviour, which might explain why it is sometimes positively related to performance (e.g., Madigan, 2019).

Beyond this, perfectionism has also been linked to psychological stress (Hewitt & Dyck, 1986). In this regard, Flett and Hewitt (2002) proposed several theoretical pathways through which perfectionism may affect stress. Two pathways are particularly relevant to the present study. The first pathway, stress perpetuation, refers to the tendency for those high in perfectionism to maintain a stressful episode via the use of maladaptive coping techniques (e.g., rumination over mistakes). The second pathway, stress enhancement, refers to the tendency for those high in perfectionism to adopt self-defeating cognitive appraisals (e.g., threat, harm, loss), resulting in the magnification of stress (e.g., overemphasising the importance of minor mistakes). These pathways may help explain why perfectionism, and particularly high levels of socially prescribed and other-oriented perfectionism, predict ill-health. However, in contrast to socially prescribed and other-oriented perfectionism, self-oriented perfectionism may buffer stress (e.g., via problem-focused coping). Despite some research supporting these pathways in athletic populations (e.g., Hill et al., 2018), no study has investigated whether perfectionism moderates the association between lifetime stressor exposure and health in elite athletes.

To address this gap in the literature, we examined (a) how lifetime stressor exposure was associated with general physical and mental health complaints among elite athletes and (b) the extent to which these associations were moderated by perfectionism (socially prescribed, self-oriented, or other-oriented). Based on the research described above, we first hypothesised that greater lifetime stressor exposure (count and severity) would be related to poorer mental and physical health among elite athletes, but that these associations would differ based on stressor timing, type, life domain, and social-psychological characteristic. Consistent with prior research (e.g., McLoughlin et al., 2021), associations between lifetime stressor exposure and health were expected to be strongest for adulthood (vs. early life) and chronic (vs. acute) stressors. Second, we hypothesised that perfectionism would moderate the relation between elite athletes’ lifetime stressor exposure and health, such that higher self-oriented perfectionism would attenuate, and greater socially prescribed and other-oriented perfectionism would strengthen, these stress-health associations.

## Method

### Participants

Participants were 110 elite athletes (64 females, 45 males, 1 preferred to self-describe) aged between 18 and 59 years old (*M*_*age*_ = 29.98 years, *SD*_*age*_ = 10.54), from a variety of sports (e.g., swimming, rugby). All participants were classified as elite because they had competed, or were currently competing, at an international or professional level (Swann et al., 2015). An *a priori* power calculation using G*Power software revealed that a minimum sample of 64 participants was required, given a medium effect of 0.30, an alpha of 0.05, and power of 0.80. The effect size was based on prior research linking lifetime stressor exposure to symptoms of depression (McLoughlin et al., 2021).

### Study design and procedure

This study used a cross-sectional design. Following institutional ethical approval (Lough-borough University Ethics Online Committee, HSPC reference: 2020-1403-1267), participants were recruited through the researchers’ existing contacts and various social media platforms (e.g., Twitter). Prior to taking part, participants read an information sheet that described the purpose of the study and informed them of their ethical rights (e.g., confidentiality, anonymity, right to withdraw). Data were collected during March and July 2020, amidst the COVID-19 pandemic. After participants had provided informed consent, data were collected through an online survey created via JISC Online Survey, which took ~45 min to complete.

### Measures

#### Cumulative lifetime stressor exposure

Lifetime stressor exposure was assessed using the Adult STRAIN (Slavich & Shields, 2018). The STRAIN is an online interview that assesses 55 major life stressors (e.g., job loss, death of a relative). For every stressor that is endorsed, follow-up questions are asked which assess stressor severity (1 = *not at all* to 5 = *extremely*), frequency (*1–5 or more* times), exposure timing (1 = *ongoing* to 7 = *over 5 years*), and duration (participants reported the number of years and/or months; Slavich & Shields, 2018). The STRAIN assesses stressors across two types (acute life events vs. chronic difficulties) and time periods (early life vs. adulthood), as well as 12 major life domains (housing, education, work, health, marital/partner, reproduction, financial, legal, other relationships, death, life-threatening situations, and possessions) and five social-psychological characteristics (interpersonal loss, physical danger, humiliation, entrapment, and role change/disruption). This study focused on the STRAIN’s two main outcomes: (1) total count of lifetime stressors experienced (range = 0–166), and (2) total severity of lifetime stressors experienced (range = 0– 265). The STRAIN has very good concurrent (*r*s = 0.15–0.62) and discriminant validity, with excellent test-retest reliability (*r*s = 0.90–0.95; Cazassa et al., 2020). Furthermore, the STRAIN has demonstrated good predictive validity in relation to various health-related outcomes, including anxiety, depression, and self-reported physical health complaints (Moseley et al., 2021; Sturmbauer et al., 2019).

#### Physical health complaints

The Physical Health Questionnaire (PHQ; Schat et al., 2005) measured physical health complaints over the past month. The PHQ consists of 14 items that assess the frequency of health complaints, including headaches, upset stomach, and colds. Eleven items (e.g., “How often have you experienced headaches?”) were scored on a 7-point Likert scale ranging from 1 (*not at all*) to 7 (*all the time*), two items (e.g., “How many times have you had minor colds that made you feel uncomfortable but didn’t keep you sick in bed or make you miss work?”) were scored on a 7-point Likert scale ranging from *0 times* to *7 + times*, and one item (“When you had a bad cold or flu, how long did it typically last?”) was scored on a 7-point Likert scale ranging from *1 day* to *7 + days*. The scores for all items were summed (range = 12–98), with higher scores indicating greater physical health complaints. The PHQ has previously demonstrated acceptable factorial validity and excellent convergent and divergent validity (Schat et al., 2005), and in this study, it demonstrated good internal consistency (*α* = 0.85).

#### Mental health complaints

The Kessler 6-Item Psychological Distress Inventory (K-6; Kessler et al., 2002) measured general mental health complaints over the past month. The K-6 consists of six items (e.g., “How often did you feel so depressed that nothing could cheer you up?”), with each item scored on a 5-point Likert scale ranging from 1 (*never*) to 5 (*very often*). The scores for all items were summed (range = 6–30), with higher scores indicating greater mental health complaints. The K-6 has previously been shown to have excellent factorial validity and strong predictive validity in relation to mental health outcomes (e.g., depression; Kessler et al., 2002). In this study, the K6 demonstrated excellent internal consistency (*ɑ* = 0.92).

#### Perfectionism

The Multidimensional Perfectionism 15-Item Scale (MPS-15; Hewitt & Flett, 1991b) measured perfectionism. The MPS-15 is comprised of three subscales: self-oriented perfectionism (e.g., “One of my goals is to be perfect in everything I do”), other-oriented perfectionism (e.g., “It doesn’t matter to me when someone close to me does not do their absolute best”), and socially-prescribed perfectionism (e.g., “Anything that I do that is less than excellent will be seen as poor work by those around me”). Each item was scored on a 7-point Likert scale ranging from 1 (*strongly disagree)* to 7 (*strongly agree*). The scores from the five items for each subscale were summed (range = 5–35), with higher scores indicating greater perfectionism. The MPS-15 is widely used and has good reliability and validity in clinical and athletic samples (Hewitt & Flett, 1991b; Stoeber, 2018). In this study, self-oriented perfectionism demonstrated good internal consistency (*α* = 0.85), while other-oriented (α = 0.71) and socially-prescribed perfectionism (*α* = 0.79) demonstrated acceptable internal consistency.

### Data analysis

Data were analysed using SPSS version 27.0. Prior to any analyses, 17 outliers (*z*-scores ≥ 3.29) were detected and removed from the dataset. Following these outlier analyses, visual inspection confirmed that all data were normally distributed. Additional checks for the other assumptions of linear regression analyses were conducted, with visual inspection of bivariate scatterplots confirming that all data were linearly related and homoscedastic.

First, to examine if total lifetime stressor count or severity predicted mental and physical health complaints, hierarchical linear multiple regression analyses were conducted. Specifically, physical and mental health complaints were entered into separate models as dependent variables, while in each model, independent variables were entered at step 1 (i.e., total lifetime stressor count or severity), and *a priori* covariates were entered at step 2 (i.e., age and gender). Next, hierarchical linear regression analyses were conducted to examine if the different stressor types (i.e., acute life events vs. chronic difficulties), exposure time periods (i.e., early-life vs. adulthood), life domains (e.g., work, death), and social-psychological characteristics (e.g., physical danger, entrapment) predicted general physical and mental health complaints, above and beyond age and gender. However, participants did not report experiencing stressors related to three life domains (i.e., education, legal, and reproduction), and thus, these stressor variants were removed from the final analyses.

Next, moderation analyses were conducted via the PROCESS SPSS custom dialog (Hayes, 2018). We used PROCESS model 1 to examine if self-oriented, other-oriented, or socially prescribed perfectionism moderated the relationship between lifetime stressor exposure (i.e., total count or severity) and physical and mental health complaints. In these analyses, in separate models, lifetime stressor count or severity [X] were entered as independent variables, physical and mental health complaints [Y] were entered as dependent variables, and the different perfectionism dimensions [W] were entered as moderating variables. A moderation model was deemed statistically significant if the 95% confidence intervals did not cross zero.

## Results

### Descriptive statistics

All descriptive statistics, including the means and standard deviations for, and correlations between, the main study variables, are shown in Table 1.

### Lifetime stressor count and health

Cumulative lifetime stressor count was significantly associated with physical (*β* = 0.34, *p* < .001) and mental (*β* = 0.49, *p* < .001) health complaints, above and beyond age and gender (Table 2). With respect to stressor type (viz. acute vs chronic), total count of acute life events was significantly associated with physical (*β* = 0.23, *p* = .024) and mental (*β* = 0.24, *p* = .018) health complaints, above and beyond age and gender. Similarly, total count of chronic difficulties was significantly associated with physical (*β* =0.24, *p* = .020) and mental (*β* = 0.36, *p* < .001) health complaints, above and beyond age and gender. With respect to the timing of stressor exposure (viz. early-life vs. adulthood), total count of early-life adversity was significantly associated with mental health complaints (*β* = 0.24, *p* = .019), above and beyond age and gender, but not physical health complaints (*β* = −0.18, *p* = .084). In contrast, total count of adulthood stressors was significantly associated with physical (*β* = 0.24, *p* = .023) and mental (*β* = 0.30, *p* = .003) health complaints, above and beyond age and gender.

**Figure 1.**
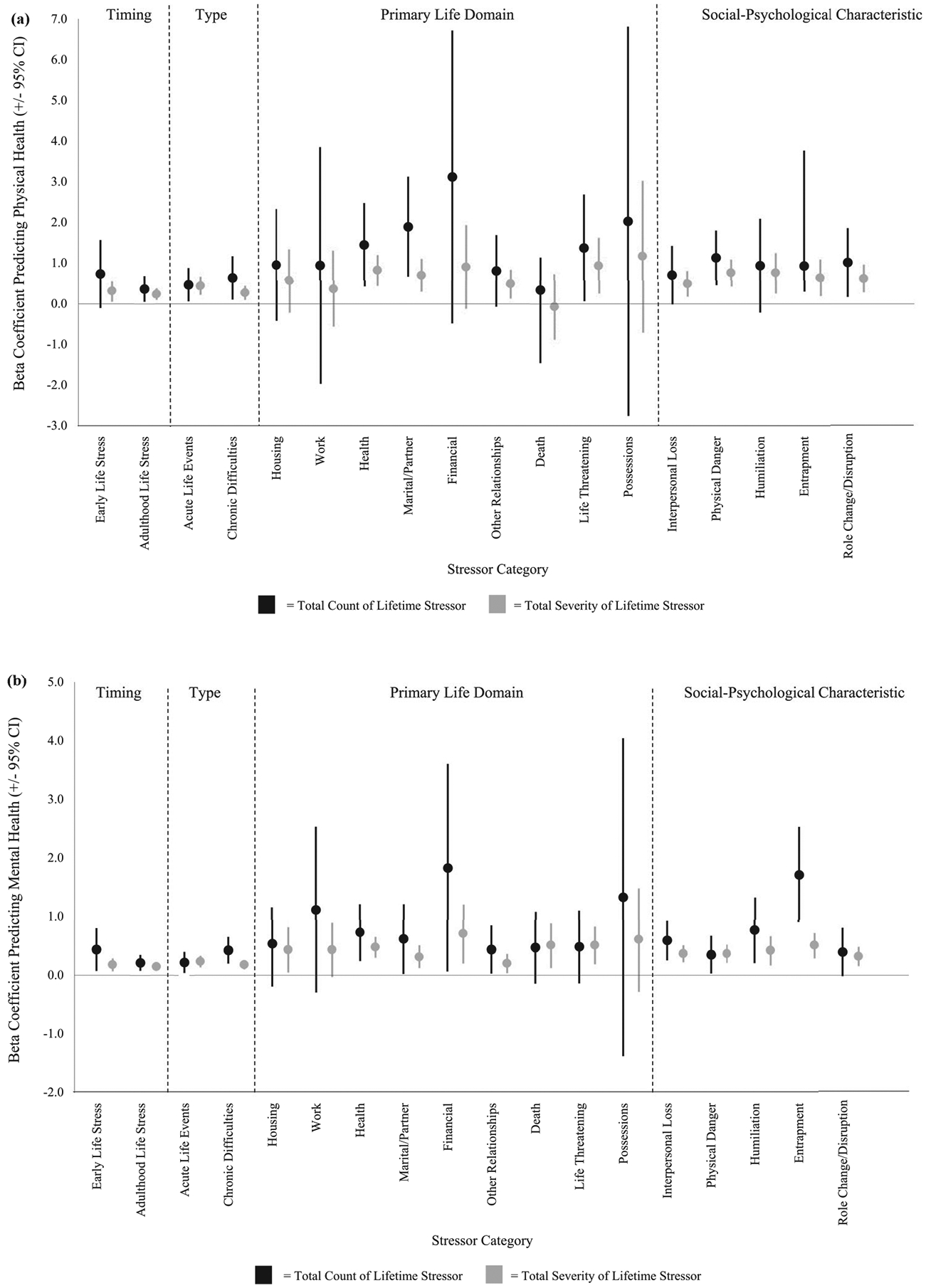
Associations between total count and severity of lifetime stress exposure and (a) physical health complaints and (b) mental health complaints, categorised by stressor timing, type, primary life domain, and social-psychological characteristic. Error bars represent 95% confidence intervals.

In terms of count, the lifetime stressor characteristics that were significantly associated with physical health complaints, while controlling for age and gender, included those involving: health (*β* = 0.26, *p* = .006), marital/partner (*β* = 0.28, *p* = .003), life-threatening situations (*β* = 0.20, *p* = .040), physical danger (*β* = 0.32, *p* = .001), entrapment (*β* = 0.22, *p* = .022), and role change (*β* = 0.23, *p* = .019). Stressors involving housing, work, financial, other relationships, death, possessions, interpersonal loss, and humiliation were not significantly associated with physical health complaints (Figure 1a). The characteristics that were significantly associated with mental health complaints, while controlling for age and gender, included those involving: health (*β* = 0.28, *p* = .004), marital/partner (*β* = 0.19, *p* = .043), financial (*β* = 0.19, *p* = .043), other relationships (*β* = 0.20, *p* = .037), interpersonal loss (*β* = 0.32, *p* = .001), physical danger (*β* = 0.21, *p* = .033), humiliation (*β* = 0.25, *p* = .008), and entrapment (*β* = 0.36, *p* < .001). Stressors involving housing, work, death, life-threatening situations, possessions, and role change were not significantly associated with mental health complaints (Figure 1b).

### Lifetime stressor severity and health

Cumulative lifetime stressor severity was significantly associated with physical (*β* = 0.38, *p* < .001) and mental (*β* = 0.57, *p* < .001) health complaints, above and beyond age and gender (Table 2). With respect to stressor type (viz. acute vs chronic), total severity of acute life events was significantly associated with physical (*β* = 0.35, *p* = .001) and mental (*β* = 0.42, *p* < .001) health complaints, above and beyond age and gender. Similarly, total severity of chronic difficulties was significantly associated with physical (*β* = 0.26, *p* = .013) and mental (*β* = 0.40, *p* < .001) health complaints, above and beyond age and gender. With respect to the timing of stressor exposure (viz. early-life vs. adulthood), total severity of early-life adversity was significantly associated with physical (*β* = 0.21, *p* = .040) and mental (*β* = 0.27, *p* = .008) health complaints, above and beyond age and gender. Comparably, total severity of adulthood stressors was significantly associated with physical (*β* = 0.30, *p* = .005) and mental (*β* = 0.42, *p* < .001) health complaints, above and beyond age and gender.

In terms of severity, the lifetime stressor characteristics that were significantly associated with physical health complaints, while controlling for age and gender, included those involving: health (*β* = 0.38, *p* < .001), marital/partner (*β* = 0.29, *p* = .002), other relationships (*β* = 0.24, *p* = .015), life-threatening situations (*β* = 0.25, *p* = .011), interpersonal loss (*β* = 0.28, *p* = .005), physical danger (*β* = 0.40, *p* < .001), humiliation (*β* = 0.27, *p* = .005), entrapment (*β* = 0.24, *p* = .010), and role change (*β* = 0.31, *p* = .001). Stressors involving housing, work, financial, death, and possessions were not significantly associated with physical health complaints (Figure 1a). The characteristics that were significantly associated with mental health complaints, while controlling for age and gender, included those involving: housing (*β* = 0.20, *p* = .037), health (*β* = 0.45, *p* < .001), marital/partner (*β* = 0.27, *p* = .004), financial (*β* = 0.25, *p* = .008), other relationships (*β* = 0.21, *p* = .036), death (*β* = 0.25, *p* = .013), life-threatening situations (*β* = 0.29, *p* = .003), interpersonal loss (*β* = 0.42, *p* < .001), physical danger (*β* = 0.41, *p* < .001), humiliation (*β* = 0.30, *p* = .002), entrapment (*β* = 0.39, *p* < .001), and role change (*β* = 0.33, *p* = .001). Stressors involving work and possessions were not significantly associated with mental health complaints (Figure 1b).

### Moderation analyses

**Figure 2.**
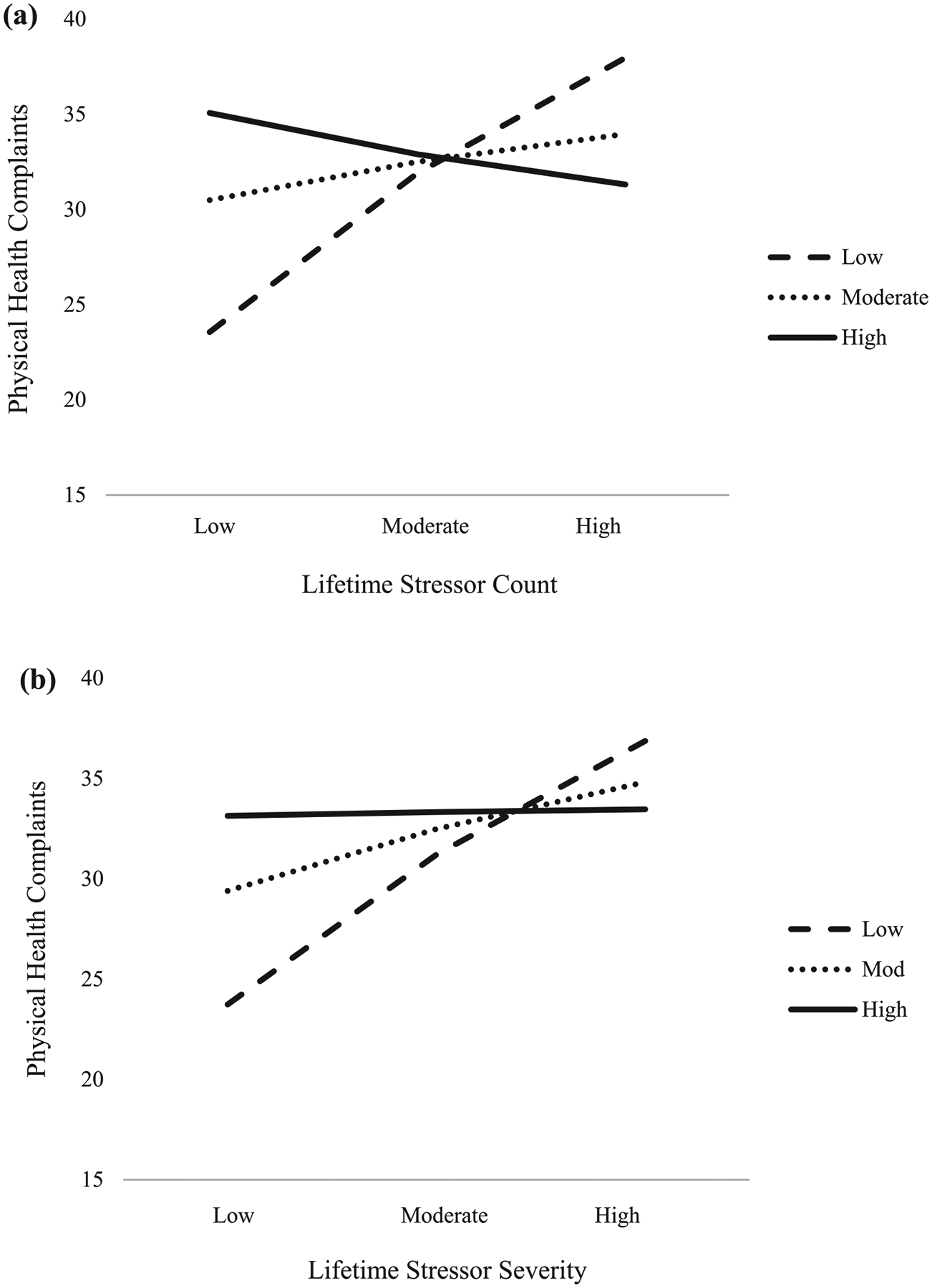
A simple slopes equation of the regression of lifetime stressor (a) count and (b) severity on physical health complaints at three levels of self-oriented perfectionism low (1 *SD* below the mean), moderate (mean), and high (1 *SD* above the mean).

No significant moderation effects were found with mental health complaints as the dependent variable, or when socially prescribed or other-oriented perfectionism were entered as moderating variables (Table 3). However, self-oriented perfectionism moderated the relation between lifetime stressor exposure and physical health complaints (Table 3). To illustrate these significant interaction effects, the association between lifetime stressor exposure, self-oriented perfectionism, and physical health complaints are depicted in Figure 2(a,b).

Self-oriented perfectionism significantly moderated the relation between lifetime stressor count and physical health complaints (*F*_(3, 89)_ = 6.16, *p* < .001, *R*^2^ = 0.17). Specifically, when self-oriented perfectionism was low, a significant positive association was observed between lifetime stressor count and physical health complaints (*b* = 0.759, 95% CI [0.400, 1.117], *t* = 4.21, *p* < .001). Yet, at the mean value of self-oriented perfectionism (*b* = 0.183, 95% CI [−0.086, 0.451], *t* = 1.35, *p* = .180), and when self-oriented perfectionism was high (*b* = −0.198, 95% CI [−0.598, 0.202], *t* = −0.98, *p* = .329), the lifetime stressor count and physical health relationship were not statistically significant. The Johnson-Neyman method illustrated that lifetime stressor count was significantly associated with physical health complaints when self-oriented perfectionism was ≤29.21. When self-oriented perfectionism exceeded 29.21, however, the lifetime stressor count-physical health association was no longer statistically significant.

Self-oriented perfectionism also significantly moderated the relation between lifetime stressor severity and physical health complaints (*F*_(3, 89)_ = 5.97, *p* < .001, *R*^2^ = 0.17). Specifically, when self-oriented perfectionism was low (*b* = 0.299, 95% CI [0.157, 0.442], *t* = 4.17, *p* < .001), and at the mean value of self-oriented perfectionism (*b* = 0.124, 95% CI [0.009, 0.238], *t* = 2.14, *p* < .0351), a significant positive association was found between lifetime stressor severity and physical health complaints. However, when self-oriented perfectionism was high, the lifetime stressor severity and physical health association was not statistically significant (*b* = 0.008, 95% CI [−0.164, 0.179], *t* = 0.09, *p* = 0.931). The Johnson-Neyman method illustrated that lifetime stressor severity was significantly associated with physical health complaints when self-oriented perfectionism was ≤30.23. When self-oriented perfectionism exceeded 30.23, however, the lifetime stressor severity-physical health association was no longer statistically significant.

## Discussion

Although greater lifetime stressor exposure has been associated with physical and mental health issues in the general population (Slavich & Shields, 2018), relatively little is known about how lifetime stressors impact the physical and mental health of elite athletes, or the factors moderating this association. To address these issues, we examined how cumulative lifetime stressor exposure was associated with general physical and mental health complaints among elite athletes, and the extent to which this association was moderated by perfectionism. We found that greater lifetime stressor exposure was associated with more general physical and mental health complaints, although the magnitude of these effects differed depending on the specific types of stressors experienced and when these stressors occurred over the life course. Collectively, these results suggest that exposure to greater and more severe lifetime stressors increases the likelihood of general physical and mental health complaints among elite athletes. These associations were robust while controlling for age and gender, which are known predictors of poorer health (e.g., Droogenbroeck et al., 2018). Furthermore, self-oriented perfectionism was found to moderate the relation between lifetime stressor exposure (count and severity) and physical health.

Although recent research has begun to show that lifetime stressor exposure may be linked to athlete mental health (e.g., McLoughlin et al., 2021), relatively little research has examined this in regard to physical health complaints (e.g., colds, coughs), particularly among elite athletes. This is surprising given that recent evidence suggests that greater stressor exposure might lead to suppressed immune function and more illness symptoms among sport performers (e.g., Drew et al., 2017). Despite this, however, research in the sports domain has predominantly focused on examining the impact of particular types of stressors on physical health (e.g., organisational stressors; Simms et al., 2020). As a result, these findings extend prior literature, importantly, demonstrating for the first time that the non-sport (personal) stressors experienced across the lifespan might influence athlete health.

With respect to stressor timing, the present results revealed that total count and severity of adulthood stressors were a marginally stronger predictor of elite athletes’ health than early life stressors. This finding is consistent with prior research conducted outside of elite sport (e.g., Lam et al., 2019), which has suggested that exposure to greater and more severe recent life stressors is more predictive of ill-health. Interestingly, the data also revealed that whereas total count of early life adversity only predicted mental health complaints, total severity of early life adversity predicted both mental and physical health complaints. Therefore, these results suggest that, for the first time, it is the severity of early life adversities that predicts physical health complaints in elite athletes rather than the total lifetime stressor count.

Turning to stressor type, total count of chronic difficulties was more strongly related to health, as compared to acute life events. This finding is consistent with prior research outside of the sports domain (e.g., Slavich et al., 2019), which has often found that chronic difficulties are particularly detrimental to health. Despite this, however, we also found that the total severity of acute life events was a relatively stronger predictor of physical and mental health complaints than the total severity of chronic difficulties. The use of qualitative research methods may be able to provide a more in-depth exploration of these contrasting findings, which are particularly important given that ill-health can interfere with social and occupational functioning (Wahlbeck, 2015). Taken together, these findings imply that stressors may have varying effects on health depending on their exposure timing (e.g., adulthood vs. early life) and type (e.g., acute life events vs. chronic difficulties).

Stressors from diverse life domains and with unique social-psychological characteristics were found to be differentially associated with physical and mental health complaints. Most notably, the lifetime stressor count and severity indices most consistently and significantly associated with health were: physical danger (e.g., being robbed at gun-point), health (e.g., on-going health problems), and interpersonal loss (e.g., close friend moves away). These results partially support prior research with elite athletes (e.g., McLoughlin et al., 2021), which found that interpersonal loss and physical danger were relatively consistent predictors of poorer mental health. These findings also advance prior research in elite sport (e.g., McLoughlin et al., 2021), demonstrating that health-related stressors can also be particularly harmful for physical and mental health. However, given that not all stressors assessed by the STRAIN were experienced (e.g., reproduction), future research should collect data from a more diverse sample to further assess the potency of these stressors.

The present study examined whether perfectionism moderated the association between lifetime stressor exposure and health among elite athletes. As hypothesised, self-oriented perfectionism significantly moderated the relation between lifetime stressor exposure (both count and severity) and physical health complaints (e.g., colds, coughs). Thus, these results suggest that self-oriented perfectionism may attenuate or buffer the positive association between lifetime stressor exposure and physical health complaints. This finding is consistent with prior research in sport, suggesting that this dimension of perfectionism is more complex than the others and can sometimes be associated with adaptive functioning (e.g., Hill et al., 2018). In contrast, no significant moderation effects were found for self-oriented perfectionism and mental health complaints. One potential explanation for this finding could be that in order to portray themselves as “perfect,” the athletes in this study may have masked symptoms of mental ill-health to hide perceived “negative” aspects of themselves (Hewitt et al., 2003). Indeed, the desire to appear perfect has been found to be associated with greater stigma and more negative attitudes towards seeking professional help for mental health difficulties (e.g., Watson et al., 2021). Furthermore, there were no significant moderation effects for other-oriented or socially-prescribed perfectionism and mental and physical health complaints. One potential explanation for this null finding could be that the sample did not have high enough levels of other-oriented (*M* = 15.56, range = 7–30) or socially-prescribed (*M* =17.03, range = 6–33) perfectionism for any moderating effects to be found.

Theoretically, the results provide support for the integrative model of lifespan stress and health (Epel et al., 2018). The integrative model suggests that personality traits and individual differences could moderate the relation between lifetime stressor exposure and long-term health outcomes (e.g., self-esteem, socially-connected individuals; Epel et al., 2018); however, it only provides a relatively limited list of personality traits. Therefore, this study represented a novel test of this proposition and advanced our understanding of the moderating role of perfectionism between lifetime stressor exposure and health. From an applied perspective, the findings might be useful in helping practitioners (e.g., coaches, sport psychologists) and sporting organisations better identify elite athletes who may be at elevated risk of physical and mental health problems and might benefit from timely intervention and adequate support (Rice et al., 2016). Indeed, by assessing elite athletes’ lifetime stressor exposure using the STRAIN, practitioners could identify athletes who have been exposed to a large amount and severe personal stressors across their lives, and particularly stressors that are chronic or have occurred recently. In doing so, practitioners might be able to prevent athletes from developing symptoms of ill-health and escalating into crisis (Schinke et al., 2018). This will be particularly important given the well-established importance of physical health, and recently emphasised position of mental health in elite sport performers (Rice et al., 2021). Furthermore, although self-oriented perfectionism moderated the relation between lifetime stressor exposure and physical health complaints, caution is required from a practical standpoint given that this dimension of perfectionism has also been linked to burnout, psychological difficulties, and performance decrements (Hill & Curran, 2015). However, given that many elite-level athletes exhibit perfectionism (e.g., Gould et al., 2002), and attribute this to their success (Madigan, 2019), practitioners should be aware of the complex role of some types of perfectionism (e.g., self-oriented) and consider tailored support for those displaying high levels of perfectionism (James & Rimes, 2018).

Several strengths and weaknesses of this study should be noted. In terms of strengths, we assessed athletes’ exposure to 55 major life stressors, including their underlying dimensions (e.g., frequency, timing, duration, and severity; Arnold & Fletcher, 2021), which has rarely been investigated. Moreover, this study is one of the first to examine how personality impacts the lifetime stressor-health relationship in sport, revealing that self-oriented perfectionism moderated the association between lifetime stressor exposure and physical health. As a result, this study highlights the potential importance of screening for lifetime stressor exposure and perfectionistic beliefs as one way to improve sport performers’ health and well-being.

With regard to limitations, the study design was cross-sectional, and directionality or causality of effects cannot be inferred. To facilitate a better understanding of how lifetime stress, perfectionism, and health affect athletes across time, future longitudinal research is recommended (Morgado et al., 2018). Second, the study used self-report measures, which could have been influenced by bias (e.g., social desirability). Future research is therefore encouraged to assess both subjective (e.g., self-report measures) and objective (e.g., salivary biomarkers) markers of psychological stress. Third, although the measurement tools used in this study have been well-validated (Schat et al., 2005; Kessler et al., 2002) and consistently used in the sporting context (e.g., Purcell et al., 2020; Turner et al., 2022), suggesting that these measures are appropriate to use in athletic populations, the present study only assessed general physical and mental health complaints (e.g., headaches, sadness). Therefore, future research could use alternative measures that may more accurately and reliably screen for mental disorders among athletes (e.g., Sport Mental Health Assessment Tool 1; Gouttebarge et al., 2020), and should account for confounding factors that may interact with perfectionism towards sporting performance (e.g.,., physical fitness, injury; Madigan et al., 2017). Fourth, this research only assessed a relatively limited number of variables (e.g., lifetime stressor exposure, health, and perfectionism), as such future research should include additional variables to further our understanding of how perfectionism moderates the lifetime stressor-health relationship. Therefore, future research could assess the theoretical pathways through which perfectionism may affect stress and health (e.g., stress perpetuation and stress enhancement; Flett & Hewitt, 2002). Finally, the present study only measured non-sport stressors and did not assess stressors experienced specifically in the sporting context (e.g., underperformance, significant injury; Sarkar & Fletcher, 2014). Indeed, while the STRAIN is a valid and reliable measure of lifetime stressor exposure, athletes encounter additional stressors to those experienced in everyday life (e.g., organisational and competitive stressors such as coach-athlete relationship difficulties and underperformance; Fletcher et al., 2006). Therefore, future research should assess sport-related stressors as well as personal stressors to improve our understanding of how lifetime stressor exposure, perfectionism, and health interact (see McLoughlin et al., 2022).

In conclusion, the present data demonstrate that exposure to greater and more severe lifetime stressors was associated with poorer health among elite athletes, but that these effects differed depending on the specific types of stressors experienced and when they occurred. Moreover, we found that self-oriented perfectionism attenuated the relation between lifetime stressor exposure and physical health complaints, suggesting that modifying such beliefs may represent a potentially useful strategy for mitigating the negative effects of stress on health. Looking forward, practitioners may benefit from being aware of the stressors that elite athletes experience and how their perfectionistic tendencies may be affecting their health in order to identify those who are at greater risk of stress-related physical and mental health problems.

## Figures and Tables

**Table 1.** Means, standard deviations, and intercorrelations for the main study variables.

Variable	Mean	*SD*	1	2	3	4	5
1. Mental health complaints	14.12	5.97	–				
2. Physical health complaints	33.13	11.78	0.61***	–			
3. Self-oriented perfectionism	28.69	4.47	−0.10	−0.07	–		
4. Other-Oriented Perfectionism	15.56	4.62	0.10	0.07	−0.04	–	
5. Socially Prescribed Perfectionism	17.03	6.54	0.06	0.10	0.32***	−0.07	–
6. Total Count of Lifetime Stressors	17.68	11.01	0.40***	0.33***	−0.01	−0.15	0.05
7. Total Severity of Lifetime Stressors	40.02	25.95	0.47***	0.36***	−0.12	−0.03	−0.03
8. Count of Acute Life Events	10.40	7.17	0.28**	0.26**	0.04	−0.16	0.06
9. Count of Chronic Difficulties	7.28	5.07	0.48***	0.36***	−0.09	−0.11	0.02
10. Count of Early Stressors	4.70	4.45	0.40***	0.24*	−0.12	−0.01	−0.01
11. Count of Adulthood Stressors	12.28	8.62	0.28**	0.28**	0.03	0.18	0.06
12. Severity of Acute Life Events	19.35	12.08	0.35***	0.32***	−0.09	−0.06	−0.02
13. Severity of Chronic Difficulties	20.80	16.03	0.51***	0.35***	−0.12	−0.01	−0.03
14. Severity of Early Stressors	12.54	11.72	0.44***	0.28**	−0.16	−0.00	−0.02
15. Severity of Adulthood Stressors	27.62	19.81	0.36***	0.31***	−0.07	−0.04	−0.02

**Table 2.** Hierarchical regression models examining if lifetime stressor (LTS) count and severity were significantly associated with (a) physical and (b) mental health complaints, both before (model 1) and after adjusting for age and gender (model 2).

	Model 1			Model 2				Model 1			Model 2		
	B	SE B	*β*	B	SE B	*β*		B	SE B	*β*	B	SE B	*β*
(a) Physical Health Complaints													
LTS Count	0.36 (0.16, 0.56)	0.10	0.33**	0.37 (0.17, 0.57)	0.10	0.34***	LTS Severity	0.17 (0.09, 0.26)	0.04	0.037***	0.18 (0.09, 0.26)	0.04	0.38***
Age				−0.18 (−0.41, 0.04)	0.11	−0.15	Age				−0.23 (−0.45, −0.03)	0.11	−0.18*
Gender				−5.11 (−9.35, −0.88)	2.13	−0.22*	Gender				−4.14 (−8.36, 0.81)	2.13	−0.18
(b) Mental Health Complaints													
LTS Count	0.25 (0.16, 0.35)	0.05	0.47***	0.27 (0.18, 0.36)	0.05	0.49***	LTS Severity	0.12 (0.08, 0.16)	0.02	0.051***	0.13 (0.09, 0.17)	0.02	0.56***
Age				−0.19 (−0.29, −0.09)	0.05	−0.31***	Age				−0.22 (−0.31, −0.12)	0.05	−0.0.36***
Gender				−1.50 (−3.40–0.40)	0.96	−0.13	Gender				−0.78 (−2.61–1.05)	0.92	−0.67

* *p* < .05;

** *p* < .01,

*** *p* < .001, two-tailed.

**Table 3.** Linear models of predictors of (a) physical and (b) mental health complaints.

	Model 1				Model 2		
	B	SE B	*t*		B	SE B	*t*
(a) Physical Health Complaints							
Constant	−19.50 (−48.21, 9.21)	14.45	−1.35	Constant	−8.98 (36.59, 18.62)	13.90	−0.65
LTS Count	3.06 (1.44, 4.68)	0.82	3.75***	LTS Severity	1.00 (0.34, 1.66)	0.33	3.03**
SOP	1.64 (0.65, 2.62)	0.49	3.30**	SOP	1.24 (0.28, 2.19)	0.48	2.58*
LTS Count × SOP	−0.10 (−0.15, −0.04)	0.03	−3.39**	LTS Severity × SOP	−0.03 (−0.05, −0.01)	0.01	−2.53*
(b) Mental Health Complaints							
Constant	−1.65 (−14.91, 11.61)	6.67	−0.25	Constant	5.39 (−6.99, 17.78)	6.23	0.87
LTS Count	0.76 (0.01, 1.51)	0.38	2.02*	LTS Severity	0.12 (−0.18, 0.41)	0.15	0.80
SOP	0.44 (−0.02, 0.89)	0.23	1.91	SOP	0.17 (−0.26, 0.59)	0.22	0.77
LTS Count × SOP	−0.02 (−0.05, 0.01)	0.01	−1.56	LTS Severity × SOP	−0.001 (−0.01, 0.01)	0.01	−0.16

Notes: *R*^2^ = 0.17,

* *p* < .05;

** *p* < .01,

*** *p* < .001, two-tailed.

Lifetime stressor (LTS) count (model 1) and severity (model 2), moderated by self-oriented perfectionism (SOP).

## Data Availability

Derived data supporting the findings of this study are available from the corresponding author on request.
